# Mitochondrial DNA variation in Parkinson’s disease: Analysis of “out-of-place” population variants as a risk factor

**DOI:** 10.3389/fnagi.2022.921412

**Published:** 2022-07-14

**Authors:** Amica C. Müller-Nedebock, Abigail L. Pfaff, Ilse S. Pienaar, Sulev Kõks, Francois H. van der Westhuizen, Joanna L. Elson, Soraya Bardien

**Affiliations:** ^1^Division of Molecular Biology and Human Genetics, Department of Biomedical Sciences, Faculty of Medicine and Health Sciences, Stellenbosch University, Cape Town, South Africa; ^2^South African Medical Research Council, Stellenbosch University Genomics of Brain Disorders Research Unit, Stellenbosch University, Cape Town, South Africa; ^3^Perron Institute for Neurological and Translational Science, Nedlands, WA, Australia; ^4^Centre for Molecular Medicine and Innovative Therapeutics, Murdoch University, Murdoch, WA, Australia; ^5^Institute of Clinical Sciences, University of Birmingham, Birmingham, United Kingdom; ^6^Human Metabolomics, North-West University, Potchefstroom, South Africa; ^7^Institute of Genetic Medicine, Newcastle University, Newcastle upon Tyne, United Kingdom

**Keywords:** Parkinsion’s disease, African ancestry, mitochondrial DNA, mtDNA, PPMI cohort, out-of-place variation, risk factor

## Abstract

Mitochondrial DNA (mtDNA), a potential source of mitochondrial dysfunction, has been implicated in Parkinson’s disease (PD). However, many previous studies investigating associations between mtDNA population variation and PD reported inconsistent or contradictory findings. Here, we investigated an alternative hypothesis to determine whether mtDNA variation could play a significant role in PD risk. Emerging evidence suggests that haplogroup-defining mtDNA variants may have pathogenic potential if they occur “out-of-place” on a different maternal lineage. We hypothesized that the mtDNA of PD cases would be enriched for out-of-place variation in genes encoding components of the oxidative phosphorylation complexes. We tested this hypothesis with a unique dataset comprising whole mitochondrial genomes of 70 African ancestry PD cases, two African ancestry control groups (*n* = 78 and *n* = 53) and a replication group of 281 European ancestry PD cases and 140 controls from the Parkinson’s Progression Markers Initiative cohort. Significantly more African ancestry PD cases had out-of-place variants than controls from the second control group (*P* < 0.0125), although this association was not observed in the first control group nor the replication group. As the first mtDNA study to include African ancestry PD cases and to explore out-of-place variation in a PD context, we found evidence that such variation might be significant in this context, thereby warranting further replication in larger cohorts.

## Introduction

Since the late 1970’s, accumulating evidence has implicated mitochondrial dysfunction as a mechanism that could underlie the characteristic loss of dopaminergic neurons in people with Parkinson’s disease (PD) (Park et al., 2018). Given the importance of mitochondria in producing adenosine triphosphate (ATP), in addition to several other essential cellular functions, compromised oxidative phosphorylation (OXPHOS) is one of many ways in which mitochondrial dysfunction could arise. Cells contain multiple copies of mitochondrial DNA (mtDNA), encoding for 13 critical components of several ATP-generating OXPHOS complexes, namely I, III, IV, and V. Following initial observations that an inhibitor of complex I could induce parkinsonism (Langston et al., 1983), mtDNA was investigated as a source of mitochondrial dysfunction in PD, almost exclusively in individuals of European and Asian descent. Despite substantial work seeking to confirm the involvement of mtDNA in PD, its precise role remains elusive and has never before been investigated in PD individuals of African ancestry (Müller-Nedebock et al., 2019).

During human evolution, the sequential accumulation of mtDNA variants in maternal lineages gave rise to stable, homoplasmic sequence variation within so-called population haplogroups that are defined by subsets of these ancient maternal variants (Van Oven and Kayser, 2009). The haplogroup association hypothesis has been a popular approach to investigate mtDNA variation in PD risk (Müller-Nedebock et al., 2019). It attempts to associate mtDNA haplogroups with a disease phenotype, suggesting that one or more common population variants may alter disease risk or the course of disease. However, past studies testing this hypothesis produced conflicting results, inconsistently associating haplogroups and/or common population variants with PD risk. These conflicting results may, at least in part, be due to methodological limitations of past studies that have left the role of population (homoplasmic) mtDNA variation in PD, unresolved (Müller-Nedebock et al., 2019). Amongst others, some past study limitations include the lack of replication cohorts as well as unaccounted-for population stratification that occurs due to mtDNA variants having a smaller effective population size than their nuclear counterparts (Salas and Elson, 2015). As such, alternative hypotheses and more standardized study designs addressing these limitations are necessary to further investigate mtDNA in PD risk.

A more recent hypothesis states that haplogroup-defining mtDNA variants, called “out-of-place” (or “local private”) variants (Kloss-Brandstätter et al., 2011), could predispose to disease when they occur on a different mtDNA lineage other than the one they define (Ji et al., 2012; Wallace, 2013; Gutiérrez Cortés et al., 2020). Empirical evidence for this hypothesis comes from studying Leber’s Hereditary Optic Neuropathy (LHON), a mtDNA-affecting disease that manifests as central vision loss (Manickam et al., 2017). For instance, one of the most common LHON-causing variants, m.11778G > A in *MT-ND4*, defines haplogroup X2p1 (van Oven, 2015) but, notably, is a confirmed cause of LHON with variable penetrance, when it occurs out-of-place on a different mtDNA background (Khan et al., 2017; Starikovskaya et al., 2019). It is postulated that haplogroup X2p1 may have additional variants that modulate the phenotypic expression of its deleterious variant or have the potential to compensate for its effects, as seen with other pathogenic human mtDNA variants in different chordate species (Queen et al., 2017; Klink et al., 2021). Furthermore, when occurring out-of-place, some common mtDNA variants may act in a deleterious synergistic manner with additional variants. This is highlighted by LHON cases, in which common mtDNA variants may work together with primary LHON mtDNA mutations to produce a more severe clinical phenotype (Achilli et al., 2012) or synergize with other common variants to cause disease in the absence of a primary pathogenic variant (Caporali et al., 2018). For instance, *MT-ND6* variants m.14258G > A [defining haplogroups U3a1a1 and H1q3 (van Oven, 2015)] and m.14582A > G [defining haplogroup H4a (van Oven, 2015)] both found in a family of haplogroup K1, were shown to cause LHON (Caporali et al., 2018). Using functional work, this study showed that cybrids carrying the mtDNA of the LHON family with these two out-of-place variants exhibited significant reductions in ATP synthesis and respiration compared to K1 haplogroup-matched control cybrids without these variants (Caporali et al., 2018).

From the evidence outlined above, there is a possibility that common population variants that are neutral or tolerable (i.e., non-pathogenic) in the haplogroups they define, may have pathogenic potential if they occur out-of-place. This can be in the setting of other haplogroups that are either unable to compensate for the presence of the variant or have variants that synergistically act together with the out-of-place variant in a harmful manner. This pathogenic potential may be less severe than that exerted by well-established disease-associated variants but could be sufficient in causing subtle bioenergetic changes that predispose to common complex disease.

Here we propose a possible role for such out-of-place haplogroup mtDNA variants in PD risk. These variants, together with additional environmental or genetic factors such as nuclear DNA (nDNA) variants and the aging-associated accumulation of somatic mtDNA variants and deletions in post-mitotic tissue (Bender et al., 2006), could compromise neurons’ OXPHOS efficiency over time and considerably so. In turn, this may facilitate the degeneration of highly energy-dependent dopaminergic neurons within the substantia nigra pars compacta, resulting in the onset and progression of PD. On these grounds, this study investigated whether more PD cases than controls harbored OXPHOS variants found outside of their usual mtDNA backgrounds. We investigated this alternative hypothesis in individuals of both African and European ancestry to determine whether mtDNA variation could play a role in PD risk.

## Methods

**FIGURE 1:**
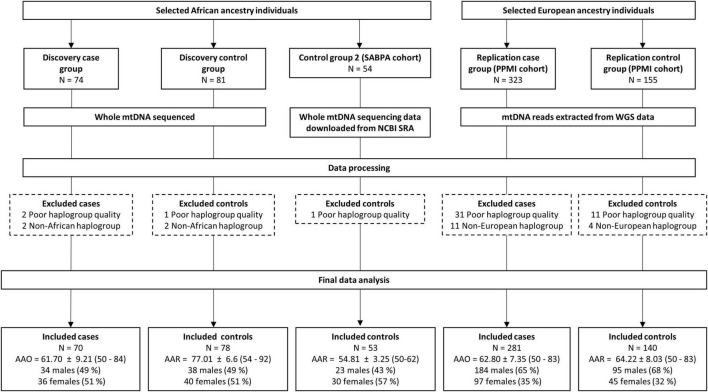
Flow diagram showing the number of African and European ancestry PD cases and controls from each group initially selected for this study, as well as the demographics of the individuals included in the final data analysis. AAO, age at onset; AAR, age at recruitment; mtDNA, mitochondrial DNA; NCBI, The National Center for Biotechnology Information; PPMI, Parkinson’s Progression Markers Initiative; SABPA, Sympathetic activity and Ambulatory Blood Pressure in Africans Study; SRA, Sequence Read Archive; WGS, whole genome sequencing.

The present study included three study groups: A discovery group of African ancestry PD cases and controls, a second African ancestry control group of selected individuals obtained from the Sympathetic Activity and Ambulatory Blood Pressure in Africans (SABPA) study cohort (Malan et al., 2015), as well as a replication study group of selected European ancestry PD cases and controls from the Parkinson’s Progression Markers Initiative (PPMI) cohort (Marek et al., 2018). Details of all individuals included in the study and final analysis are provided in Figure 1 and Supplementary Table 1.

### Discovery group study participants

A previously recruited group of unrelated, African ancestry individuals (self-identifying as “Black”), consisting of 74 individuals diagnosed with PD and 81 aged controls, were selected as the discovery group. Given that several PD-causing nuclear genetic determinants have been associated with an earlier age at onset (Li et al., 2021), we only included late-onset PD cases (age at PD onset ≥ 50 years). All controls were older than 50 years of age and did not have PD at the time of recruitment.

Cases were recruited from the Movement Disorders Clinic at Tygerberg Hospital in Cape Town, South Africa, and from various neurology clinics around the country, while controls were recruited from blood donation clinics in South Africa’s Western Cape Province and non-neurology clinics in the Eastern Cape province. All cases were examined by a Movement Disorder specialist to confirm they met the United Kingdom Parkinson’s Disease Society Brain Bank Research criteria for a PD diagnosis (Gibb and Lees, 1988). All study participants provided written consent for this study and the study was granted ethical clearance by the Health Research Ethics Committee at Stellenbosch University (protocol numbers: 2002/C059 and S20/01/010). The study conformed with the ethical standards of the World Medical Association Declaration of Helsinki.

### The second control group and replication group study participants

A limitation of past studies investigating mtDNA variation in PD was the lack of a second control group or a replication cohort. The inclusion of either is recommended in mtDNA association studies to detect population substructure, which can result in false-positive associations, affecting the validity of results if not taken into consideration (Salas and Elson, 2015). Consequently, we included a second control group as well as a replication study group consisting of cases and controls, 50 years of age or older. The former consisted of African ancestry individuals for whom whole mtDNA sequencing data was publicly available (BioProject accession number: PRJNA403942). These individuals were from the SABPA study cohort, recruited from the North-West province of South Africa. Further details on participant recruitment, sample, and data collection methods have been published elsewhere (Malan et al., 2015).

Given that a replication cohort consisting of African ancestry PD cases and controls was not available, our replication study group consisted of European ancestry individuals (self-identifying as “White”) from the PPMI cohort, for whom whole-genome sequencing data was available. Demographics and study recruitment of these individuals were previously outlined elsewhere (Marek et al., 2018). For our replication study group, we included PPMI individuals diagnosed with PD (age at onset of disease ≥ 50 years) and aged controls 50 years or older, to match the discovery group.

### Targeted mitochondrial DNA sequencing of African ancestry (discovery group) Parkinson’s disease cases and controls

Genomic DNA, isolated from whole blood of the previously recruited African ancestry study participants was used for mtDNA sequencing. Briefly, the mitochondrial genomes of the selected study participants were sequenced using the Precision ID mtDNA Whole Genome Panel (Thermo Fisher Scientific, Waltham, United States) on the Ion S5 System (Thermo Fisher Scientific), in accordance with the manufacturer’s protocol. Sequencing was performed at the Central Analytical Facilities (Stellenbosch University, South Africa). The mitochondrial genomes of the 155 individuals were successfully sequenced to an average coverage depth of 2,992 ± 589x (1,276–4,240x). Further details on the mtDNA sequencing process are provided in Supplementary Material. After the sequencing runs, the Ion Torrent Suite Software (Thermo Fisher Scientific) automatically performed sequence trimming, quality filtering, and read alignment. Reads were aligned to the revised Cambridge reference sequence (rCRS; NC_012920.1) to produce BAM files for the 74 PD cases and 81 controls.

### FASTQ file preparation for the sympathetic activity and ambulatory blood pressure in Africans and Parkinson’s progression markers initiative study groups

The whole mtDNA sequences from the SABPA cohort were previously deposited in the Sequence Read Archive (SRA) of the National Center for Biotechnology Information (NCBI), under the BioProject accession number PRJNA403942.^1^ These genomes were sequenced at a mean coverage of ∼1,800x from two overlapping long-range PCR products using the Ion PGM system (Thermo Fisher Scientific) (Venter et al., 2017). The SRA Toolkit (NCBI)^2^ was used to download the whole mtDNA sequences of the 54 African ancestry SAPBA individuals aged 50 years or older via the command line. The downloaded files were subsequently converted to a usable FASTQ single-end format using the NCBI SRA Toolkit.

For the PPMI study participants, high coverage whole genome sequencing data generated on the HiSeq X platform (Illumina, San Diego, United States), were obtained upon application from the PPMI.^3^ These data were in BAM file format that had been aligned to the GRCh38 reference genome using BWA (Li and Durbin, 2009). Reads mapping to the mitochondrial genome were extracted using SAMtools (Li et al., 2009) for 323 cases and 155 controls aged 50 years or older. The resulting BAM files were then sorted by name and converted to paired-end FASTQ files, ready for realignment to the rCRS. The mean coverage of the mtDNA genomes of PPMI individuals was 1,097x ± 644x (338–4,879).

### Data processing and analysis

**FIGURE 2:**
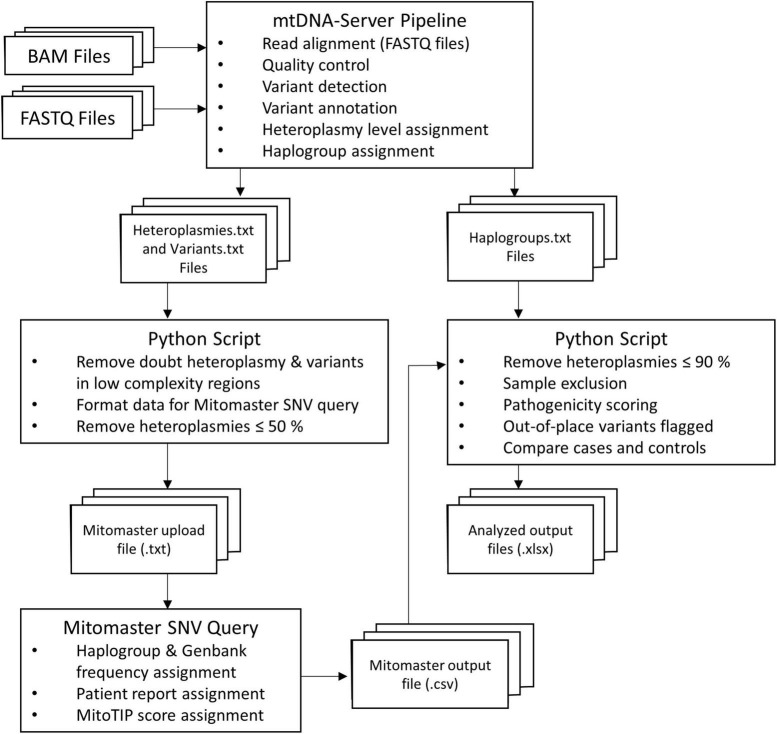
Flow diagram outlining the data analysis process of the present study. The mtDNA-Server pipeline was used to process the BAM/FASTQ files containing mtDNA reads of the study participants. A Python script was used to filter mtDNA-Server variant files and prepare new variant files that could be uploaded to Mitomaster. Annotated variant files from Mitomaster, together with the previously generated mtDNA-Server haplogroup files were then used as input for a second Python script that tested the out-of-place variant hypothesis.

A workflow summary of the data processing and analysis is provided in Figure 2. BAM and FASTQ files of all individuals were processed using the mtDNA-Server pipeline (Weissensteiner et al., 2016). Its workflow includes read alignment (for FASTQ files) to the rCRS, variant annotation, haplogroup assignment using HaploGrep (Kloss-Brandstätter et al., 2011), and describing the proportion of variant molecules (heteroplasmy). Output files are text (.txt) files that contain all study participants’ assigned haplogroups, annotated mtDNA variants, and the homoplasmy or heteroplasmy level of these variants. A Python (v. 3.8.0)^4^ script was written to create a Mitomaster upload file, containing variants called by the mtDNA-Server pipeline in the format required by the Mitomaster single nucleotide variant (SNV) query.^5^ The script filtered out variants in homopolymer regions and doubt heteroplasmies (denoted as type-2 and -3 heteroplasmies), together with variants with a heteroplasmy level of 50% or below. The generated Mitomaster upload files were uploaded to the Mitomaster SNV query page that generated .csv output files containing Genbank and haplogroup frequencies of all variants, MitoTIP scores (Sonney et al., 2017), and information on whether variants were associated with a disease (e.g., LHON).

A second Python script was written to test the out-of-place hypothesis put forward here, requiring the mtDNA-Server output files and the generated Mitomaster files as input. It was run three times to compare: (1) African ancestry discovery group cases vs. controls, (2) discovery cases vs. the SABPA second control group, and (3) European ancestry PPMI replication group cases vs. controls. The script excluded variants with a heteroplasmy level of 90% or below (i.e., only operationally homoplasmic variants were included in the analysis, and low-level heteroplasmic variants were not considered for this hypothesis). It additionally excluded samples with a poor haplogroup quality score (≤90%), and individuals with a non-African haplogroup (run 1 and 2) or non-European haplogroup (run 3). A list of all identified variants (>90% heteroplamsy) per individual can be found in Supplementary Table 2. We defined African haplogroups as those belonging to African maternal lineages L0, L1, L2, L3, L4, L5, and L6. European haplogroups were ones belonging to classical European maternal lineages, namely H, V, U, K, T, J, I, W, and X. The script additionally assigned pre-computed pathogenicity prediction scores from MitImpact v. 3.06 (APOGEE and Mtoolbox scores) (Castellana et al., 2015) as well as MutPred (according to Pereira et al., 2011) to non-synonymous variants. It also identified out-of-place variants (described as “local private mutations” according to HaploGrep) (Kloss-Brandstätter et al., 2011) and compared the number of cases and controls from each of the maternal lineages, with and without these out-of-place variants by using Fisher’s exact tests. More specifically, the script compared the number of cases and controls carrying: (1) out-of-place mtDNA variants in the genes encoding components of the OXPHOS chain, (2) synonymous out-of-place variants, (3) non-synonymous out-of-place variants, and (4) scored non-synonymous out-of-place variants in these genes. Bonferroni correction was used to adjust significance levels to 0.0125 for these comparisons. Due to the modest sample sizes, statistical comparisons for individual OXPHOS complexes, genes and variants between cases and controls were not done. Fisher’s exact tests (with Bonferroni correction) were also used for testing whether cases and controls differed significantly in terms of their maternal ancestries. Statistical analyses were performed using SPSS software v. 27 (IBM, Armonk, NY, United States). Graphs presented here were generated using GraphPad Prism v. 5 (GraphPad Software, San Diego, California, United States) and Python (v. 3.8.0). Python scripts used for data analysis can be found on GitHub.^6^

## Results

### Pathogenic and unreported mitochondrial DNA variation in all study participants

A summary of the mean number of variants called per person for each of the different study group cases and controls is shown in Supplementary Table 3. Overall, the African ancestry individuals had more mtDNA variants called against the rCRS than those of European ancestry. These findings were to be expected, given that the phylogenetic distance between the rCRS (a European ancestry sequence belonging to haplogroup H2a2a1) and the African ancestry sequences is much greater than the distance between the European ancestry sequences and the rCRS.

Prior to testing the out-of-place hypothesis, we examined all cases and controls for the presence of confirmed pathogenic variants (according to Mitomap; Lott et al., 2013) and variants that are not present (i.e., unreported) in Mitomap, the genome aggregation database (gnomAD) v. 3.1 (Karczewski et al., 2020), and HelixMTdb.^7^ A total of four study participants carried variants that had previously been associated with a disease (Table 1). One African ancestry PD case [age at onset (AAO) = 64; haplogroup L3e1b2] harbored a previously unreported variant (m.12132C > G) in *MT-ND4*, predicted to be benign by the pathogenicity scoring tools used in our analysis. None of the other African ancestry individuals nor the PPMI individuals carried unreported variants. Moreover, none of the study participants had tRNA variants that were predicted to be deleterious.

**TABLE 1:** Study participants carrying mitochondrial DNA variants previously confirmed to be associated with a disease.

Variant (Associated disease)	Haplogroup-defining variant (Haplogroup)	Individuals carrying the variant	Study group
m.11778G > A (LHON)	Yes (X2p1)	African ancestry female control (AAR = 85; haplogroup L3e2b1a2)	Discovery
		European ancestry female PD case (AAO = 59; haplogroup U5a1a1)	Replication
m.14484T > C (LHON)	Yes (Q3b)	European ancestry male control (AAR = 72; haplogroup H3c2b1)	Replication
m.1555A > G (Deafness)	No	European ancestry female control (AAR = 57; haplogroup U4b1b1)	Replication

AAO, age at onset; AAR, age at recruitment LHON, Leber’s Hereditary Optic Neuropathy; PD, Parkinson’s disease.

### Haplogroup distribution

**FIGURE 3:**
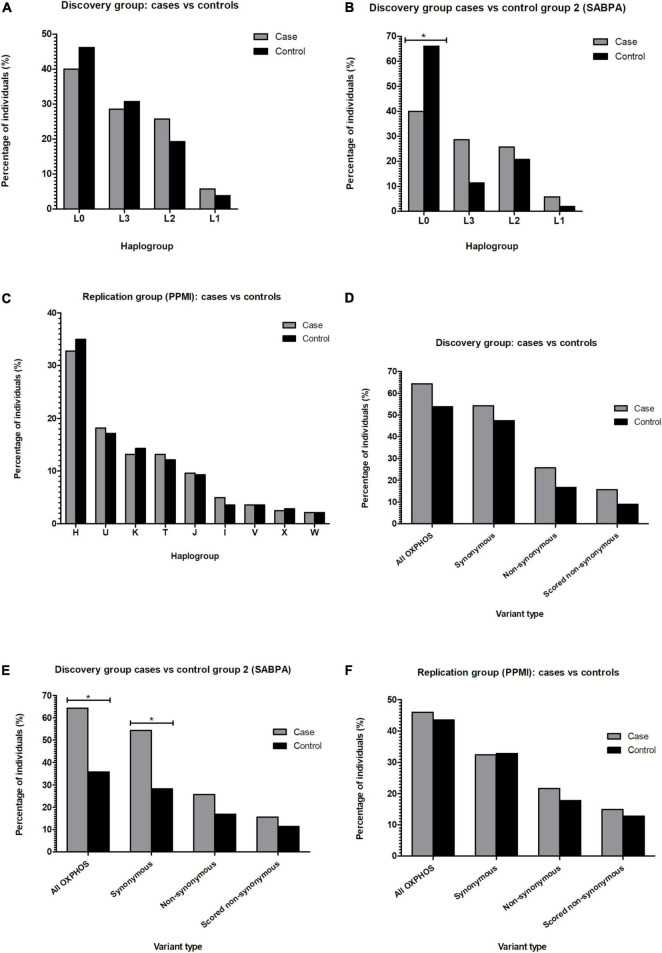
Bar graphs showing the percentage of PD cases and controls from African **(A,B)** or European **(C)** maternal lineages identified in the study, as well as the percentage of PD cases vs. controls carrying out-of-place mtDNA variants (**D**–**F**). *P < 0.0125. mtDNA, mitochondrial DNA; OXPHOS, oxidative phosphorylation; PPMI, Parkinson’s Progression Markers Initiative; SABPA, Sympathetic activity and Ambulatory Blood Pressure in Africans Study.

The African ancestry study participants belonged to lineages L0, L1, L2, or L3 (Supplementary Table 4), and none were found to belong to African lineages L4–6. While the haplogroup frequencies of the discovery group cases and controls did not differ significantly (Figure 3A), significantly more SABPA controls from the second control group (66%) than PD cases from the discovery group (46%) belonged to haplogroups of the L0 lineage (*P* < 0.0125) (Figure 3B).

All European lineages (H, I, J, K, T, V, W, X, and U) were present in the replication (PPMI) group (Supplementary Table 4). As seen in the discovery group, cases and controls of the replication group did not differ significantly in terms of their assigned lineages (Figure 3C).

### Investigating out-of-place mitochondrial DNA variants in Parkinson’s disease risk

When testing the out-of-place hypothesis in our African ancestry discovery group, we observed no significant differences between the number of cases and controls carrying out-of-place OXPHOS variants (Figure 3D; *P* > 0.0125). However, when using the second African ancestry (SABPA) control group, we found that significantly more discovery group PD cases than SABPA controls carried out-of-place variants in genes encoding components of the OXPHOS complexes (Figure 3E; *P* < 0.0125). We again found significantly more discovery cases than controls from the SABPA control group (*P* < 0.0125) harboring synonymous out-of-place variants in their OXPHOS genes (Figure 3E), when examining synonymous and non-synonymous variants separately.

Using a larger, independent group of PD cases and controls from the PPMI cohort as a replication study group we observed no significant differences between the number of cases and controls with these out-of-place OXPHOS variants (Figure 3F; *P* > 0.0125).

## Discussion

To date, studies investigating homoplasmic mtDNA variation in PD have largely been limited to individuals of European ancestry and have mostly employed the haplogroup association approach. However, conflicting findings emanating from these past studies have highlighted the need for alternative approaches to investigate mtDNA variation in PD risk (Müller-Nedebock et al., 2019).

The present study explored the out-of-place variant hypothesis that investigated whether carrying haplogroup-defining OXPHOS variants found out-of-place on a different maternal lineage could be associated with PD. Importantly, unlike previous mtDNA PD investigations, we included individuals of African and European ancestry in our study. We found significantly more African ancestry PD cases (discovery group cases) harboring out-of-place OXPHOS variants compared to controls from the SABPA cohort, thereby supporting a possible association between these variants and PD. Examining synonymous and non-synonymous out-of-place variants separately revealed that significantly more of these cases than controls harbored synonymous variants, possibly hinting at the pathogenic potential of these variants in particular, when found outside of their usual haplogroup. Although synonymous variants were previously thought to be benign, it is now well established that this is not the case (Sauna and Kimchi-Sarfaty, 2011), with recent evidence implying that synonymous variants are almost just as important as non-synonymous ones in causing disease (Shen et al., 2022). It would therefore be interesting to test for a possible functional effect of out-of-place synonymous variants in future cybrid studies using mDNA derived from individuals with and without these variants, but otherwise identical mtDNA backgrounds.

Importantly, our findings extend the possible role of out-of-place variants in disease, from mtDNA-related LHON to PD—a more common and complex disease with a strong link to mitochondrial dysfunction. We speculate that these variants could cause subtle bioenergetic changes that over time, together with additional PD risk factors, contribute to mitochondrial dysfunction. This in turn could culminate in disease onset. Consequently, it is tempting to speculate that out-of-place variants could also predispose to complex diseases other than PD that have links to mitochondrial dysfunction.

To address some limitations of past mtDNA association studies, our study design included two control groups, as well as a replication group, whilst we also corrected for multiple testing. Moreover, to increase the reproducibility between future studies, we used publicly available tools to process our sequencing data and wrote reproducible code that tests the hypothesis put forward here. However, one limitation that we could not address was the relatively small sample size. Our modest sample sizes may explain why we did not observe a significant difference between the number of cases and controls with out-of-place OXPHOS variants across all three of our study groups. We, therefore, propose that more studies with larger sample sizes, including individuals of African ancestry, may be needed to reproducibly associate out-of-place mtDNA variation with PD. In addition to late-onset PD cases, future studies could also consider including early onset PD cases to increase their sample sizes. Another important issue that was addressed in this study, was population stratification (i.e., allele-frequency differences between subpopulations in a population due to different ancestries) that has likely caused false-positive associations between mtDNA and PD in the past. We hypothesized that our approach would not be prone to this type of stratification, given that out-of-place variants will be less frequent, and by definition occurring sporadically across the phylogeny.

It should, however, be noted that there was also a significant difference between the maternal lineages of our discovery PD cases and the SABPA control group (Figure 3E), likely reflecting the differing haplogroup distributions of the geographic locations from which the study groups were recruited within South Africa. This observation in turn suggested that our approach might be affected by a form of stratification. It may be that mtDNA background influences the development of mtDNA mutagenesis (Wei et al., 2017) and that some mtDNA lineages are more evolutionary robust than others; i.e., some mtDNA lineages might acquire, retain or tolerate additional variants more or more often than others (Klink et al., 2021). This could enable *de novo* out-of-place and/or pathogenic variants to occur more frequently on certain mtDNA lineages. However, further research into whether or not this occurs will be needed.

### The unpredictable effects of mitochondrial DNA variation

Notably, four of our study participants carried mtDNA variants previously confirmed to be associated with LHON or deafness. This is in line with findings from previous studies that have reported these and other pathogenic mtDNA variants in the general population at varying levels of heteroplasmy (4.4–100.0%) (Elliott et al., 2008; Venter et al., 2019; Marshall et al., 2020). To the best of our knowledge, none of the individuals carrying these mtDNA variants were affected by either of these conditions, reinforcing the well-accepted notion that additional genetic (i.e., mtDNA and nDNA backgrounds) and environmental factors [e.g., exposure to aminoglycoside antibiotics that induce non-syndromic deafness in carriers of the m.1555A > G variant (Ou et al., 2018)] may play a role in the expression of a mtDNA-associated disease phenotype (Khan et al., 2017; Wei et al., 2017). These observations once again highlight the difficulty of assessing the impact that a mtDNA variant might have in different individuals. Overall, studying mtDNA variation as a contributor to disease risk is notoriously challenging (McCormick et al., 2020) given the genome’s unique features of maternal inheritance, the contextual effects of haplogroups (Khan et al., 2017; Starikovskaya et al., 2019) as well as heteroplasmy and the threshold effect. For instance, the same mtDNA variant (e.g., m.3395A > G) can predispose to multiple different diseases at varying levels of heteroplasmy (Gutiérrez Cortés et al., 2020). Furthermore, tissue-specific differences in mtDNA replication, variant accumulation, gene expression, and heteroplasmy regulation may exist, all of which further contribute to the difficulty of predicting the effect of mtDNA variants.

### Unreported mitochondrial DNA variation

The fact that only one of our study participants (an African PD case) carried a previously unreported variant may reflect the progress made in terms of capturing human mtDNA variation across different populations (van Oven, 2015). However, African genomes are still largely underrepresented in public genomic databases and the currently reported population variant frequencies do not reflect the actual population frequencies (McCormick et al., 2020). This is problematic, given that these frequencies are used as a criterion to interpret the pathogenicity of both nDNA and mtDNA variants. Therefore, the underrepresentation of non-European ancestry individuals in clinically relevant databases could limit diagnostic accuracy for underrepresented population groups (Bentley et al., 2020). Consequently, genetic studies that include African cohorts are important for making genomic databases more representative of all ancestries and for updating the frequencies at which individual variants are found across understudied populations. Once these databases become more representative of all ancestries, it will become possible to more accurately assess the role of both common and rare population variants in PD risk within currently understudied populations. This will enable future studies to investigate additional alternative hypotheses, such as the common disease rare variant hypothesis (Schork et al., 2009) in PD, using both nDNA and mtDNA variation.

## Conclusion

To conclude, this was the first investigation to sequence and study whole mitochondrial genomes of African ancestry PD cases and to explore out-of-place variation in the PD context. We found evidence within our unique dataset that such variants might be significant in this context, illustrating the utility of African genomes to shed light on the genetic underpinnings of human disease.

## Data availability statement

The original contributions presented in this study are included in the article/Supplementary Material, further inquiries can be directed to the corresponding author/s.

## Ethics statement

The studies involving human participants were reviewed and approved by the Health Research Ethics Committee at Stellenbosch University (protocol numbers: 2002/C059 and S20/01/010). The patients/participants provided their written informed consent to participate in this study.

## Author contributions

AM-N, SB, FW, and JE: conceptualization. AM-N and JE: formal analysis. AM-N and SB: funding acquisition. AM-N and AP: investigation. AM-N, SB, FW, and SK: resources. AM-N: software and visualization. AM-N, AP, SB, and JE: methodology. AM-N, SB, IP, and JE: writing—original draft. AM-N, SB, FW, JE, IP, AP, and SK: writing—review and editing. All authors contributed to the article and approved the submitted version.

## Conflict of interest

The authors declare that the research was conducted in the absence of any commercial or financial relationships that could be construed as a potential conflict of interest.

## Publisher’s note

All claims expressed in this article are solely those of the authors and do not necessarily represent those of their affiliated organizations, or those of the publisher, the editors and the reviewers. Any product that may be evaluated in this article, or claim that may be made by its manufacturer, is not guaranteed or endorsed by the publisher.

## Footnotes

1
https://www.ncbi.nlm.nih.gov/bioproject/403942

2
https://trace.ncbi.nlm.nih.gov/Traces/sra/sra.cgi?view=software

3
www.ppmi-info.org/data

4
http://www.python.org

5
https://mitomap.org/mitomaster/index_snvs.cgi

6
https://github.com/amicamuller/mtDNA

7
https://www.helix.com/pages/mitochondrial-variant-database

## References

[B1] Achilli A. Iommarini L. Olivieri A. Pala M. Kashani B. H. Reynier P. (2012). Rare Primary Mitochondrial DNA Mutations and Probable Synergistic Variants in Leber’s Hereditary Optic Neuropathy. *PLoS One* 7:e42242. 10.1371/journal.pone.0042242 22879922 PMC3411744

[B2] Bender A. Krishnan K. J. Morris C. M. Taylor G. A. Reeve A. K. Perry R. H. (2006). High levels of mitochondrial DNA deletions in substantia nigra neurons in aging and Parkinson disease. *Nat. Genet.* 38:515. 10.1038/ng1769 16604074

[B3] Bentley A. R. Callier S. L. Rotimi C. N. (2020). Evaluating the promise of inclusion of African ancestry populations in genomics. *NPJ Genomic Med.* 5:5. 10.1038/s41525-019-0111-x 32140257 PMC7042246

[B4] Caporali L. Iommarini L. La Morgia C. Olivieri A. Achilli A. Maresca A. (2018). Peculiar combinations of individually non-pathogenic missense mitochondrial DNA variants cause low penetrance Leber’s hereditary optic neuropathy. *PLoS Genet.* 14:e1007210. 10.1371/journal.pgen.1007210 29444077 PMC5828459

[B5] Castellana S. Rónai J. Mazza T. (2015). MitImpact: an exhaustive collection of pre-computed pathogenicity predictions of human mitochondrial non-synonymous variants. *Hum. Mutat.* 36 E2413–E2422. 10.1002/humu.22720 25516408

[B6] Elliott H. R. Samuels D. C. Eden J. A. Relton C. L. Chinnery P. F. (2008). Pathogenic Mitochondrial DNA Mutations Are Common in the General Population. *Am. J. Hum. Genet.* 83 254–260. 10.1016/j.ajhg.2008.07.004 18674747 PMC2495064

[B7] Gibb W. R. G. Lees A. J. (1988). A comparison of clinical and pathological features of young-and old-onset Parkinson’s disease. *Neurology* 38 1402–1402. 10.1212/wnl.38.9.1402 3412587

[B8] Gutiérrez Cortés N. Pertuiset C. Dumon E. Börlin M. Da Costa B. Le Guédard M. (2020). Mutation m.3395A>G in MT-ND1 leads to variable pathologic manifestations. *Hum. Mol. Genet.* 29 980–989. 10.1093/hmg/ddaa020 32011699

[B9] Ji F. Sharpley M. S. Derbeneva O. Alves L. S. Qian P. Wang Y. (2012). Mitochondrial DNA variant associated with Leber hereditary optic neuropathy and high-altitude Tibetans. *Proc. Natl. Acad. Sci. U. S. A.* 109 7391–7396. 10.1073/pnas.1202484109 22517755 PMC3358837

[B10] Karczewski K. J. Francioli L. C. Tiao G. Cummings B. B. Alföldi J. Wang Q. (2020). The mutational constraint spectrum quantified from variation in 141,456 humans. *Nature* 581 434–443. 10.1038/s41586-020-2308-7 32461654 PMC7334197

[B11] Khan N. A. Govindaraj P. Soumittra N. Sharma S. Srilekha S. Ambika S. (2017). Leber’s Hereditary Optic Neuropathy–Specific Mutation m.11778G>A Exists on Diverse Mitochondrial Haplogroups in India. *Invest. Ophthalmol. Vis. Sci.* 58 3923–3930. 10.1167/iovs.16-20695 28768321

[B12] Klink G. V. O’Keefe H. Gogna A. Bazykin G. A. Elson J. L. (2021). A broad comparative genomics approach to understanding the pathogenicity of Complex I mutations. *Sci. Rep.* 11:19578. 10.1038/s41598-021-98360-7 34599203 PMC8486755

[B13] Kloss-Brandstätter A. Pacher D. Schönherr S. Weissensteiner H. Binna R. Specht G. (2011). HaploGrep: a fast and reliable algorithm for automatic classification of mitochondrial DNA haplogroups. *Hum. Mutat.* 32 25–32. 10.1002/humu.21382 20960467

[B14] Langston J. W. Ballard P. Tetrud J. W. Irwin I. (1983). Chronic Parkinsonism in humans due to a product of meperidine-analog synthesis. *Science* 219 979–980. 10.1126/science.6823561 6823561

[B15] Li B. Zhao G. Zhou Q. Xie Y. Wang Z. Fang Z. (2021). Gene4PD: a Comprehensive Genetic Database of Parkinson’s Disease. *Front. Neurosci.* 15:679568. 10.3389/fnins.2021.679568 33981200 PMC8107430

[B16] Li H. Durbin R. (2009). Fast and accurate short read alignment with Burrows-Wheeler transform. *Bioinforma. Oxf. Engl.* 25 1754–1760. 10.1093/bioinformatics/btp324 19451168 PMC2705234

[B17] Li H. Handsaker B. Wysoker A. Fennell T. Ruan J. Homer N. (2009). The Sequence Alignment/Map format and SAMtools. *Bioinformatics* 25 2078–2079. 10.1093/bioinformatics/btp352 19505943 PMC2723002

[B18] Lott M. T. Leipzig J. N. Derbeneva O. Xie H. M. Chalkia D. Sarmady M. (2013). mtDNA Variation and Analysis Using Mitomap and Mitomaster. *Curr. Protoc. Bioinforma.* 44 1.23.1–26. 10.1002/0471250953.bi0123s44 25489354 PMC4257604

[B19] Malan L. Hamer M. Frasure-Smith N. Steyn H. S. Malan N. T. (2015). Cohort Profile: sympathetic activity and Ambulatory Blood Pressure in Africans (SABPA) prospective cohort study. *Int. J. Epidemiol.* 44 1814–1822. 10.1093/ije/dyu199 25344943 PMC4689997

[B20] Manickam A. H. Michael M. J. Ramasamy S. (2017). Mitochondrial genetics and therapeutic overview of Leber’s hereditary optic neuropathy. *Indian J. Ophthalmol.* 65 1087–1092. 10.4103/ijo.IJO_358_17 29133631 PMC5700573

[B21] Marek K. Chowdhury S. Siderowf A. Lasch S. Coffey C. S. Caspell-Garcia C. (2018). The Parkinson’s progression markers initiative (PPMI) – establishing a PD biomarker cohort. *Ann. Clin. Transl. Neurol.* 5 1460–1477. 10.1002/acn3.644 30564614 PMC6292383

[B22] Marshall C. Sturk-Andreaggi K. Ring J. D. Dür A. Parson W. (2020). Pathogenic Variant Filtering for Mitochondrial Genome Haplotype Reporting. *Genes* 11:1140. 10.3390/genes11101140 32998193 PMC7599696

[B23] McCormick E. M. Lott M. T. Dulik M. C. Shen L. Attimonelli M. Vitale O. (2020). Specifications of the ACMG/AMP standards and guidelines for mitochondrial DNA variant interpretation. *Hum. Mutat.* 41 2028–2057. 10.1002/humu.24107 32906214 PMC7717623

[B24] Müller-Nedebock A. C. Brennan R. R. Venter M. Pienaar I. S. van der Westhuizen F. H. Elson J. L. (2019). The unresolved role of mitochondrial DNA in Parkinson’s disease: an overview of published studies, their limitations, and future prospects. *Neurochem. Int.* 129:104495. 10.1016/j.neuint.2019.104495 31233840 PMC6702091

[B25] Ou Y. H. Chen A. W. G. Fan J. Y. Cheng W. L. Lin T. T. Chen M. K. (2018). Aminoglycoside-associated nonsyndromic deafness and speech disorder in mitochondrial A1555G mutation in a family. *Medicine* 97:e12878. 10.1097/MD.0000000000012878 30335006 PMC6211905

[B26] Park J. S. Davis R. L. Sue C. M. (2018). Mitochondrial Dysfunction in Parkinson’s Disease: new Mechanistic Insights and Therapeutic Perspectives. *Curr. Neurol. Neurosci. Rep.* 18:21. 10.1007/s11910-018-0829-3 29616350 PMC5882770

[B27] Pereira L. Soares P. Radivojac P. Li B. Samuels D. C. (2011). Comparing phylogeny and the predicted pathogenicity of protein variations reveals equal purifying selection across the global human mtDNA diversity. *Am. J. Hum. Genet.* 88 433–439. 10.1016/j.ajhg.2011.03.006 21457906 PMC3071914

[B28] Queen R. A. Steyn J. S. Lord P. Elson J. L. (2017). Mitochondrial DNA sequence context in the penetrance of mitochondrial t-RNA mutations: a study across multiple lineages with diagnostic implications. *PLoS One* 12:e0187862. 10.1371/journal.pone.0187862 29161289 PMC5697862

[B29] Salas A. Elson J. L. (2015). Mitochondrial DNA as a risk factor for false positives in case-control association studies. *J. Genet. Genomics* 42 169–172. 25953355 10.1016/j.jgg.2015.03.002

[B30] Sauna Z. E. Kimchi-Sarfaty C. (2011). Understanding the contribution of synonymous mutations to human disease. *Nat. Rev. Genet.* 12 683–691. 10.1038/nrg3051 21878961

[B31] Schork N. J. Murray S. S. Frazer K. A. Topol E. J. (2009). Common vs. rare allele hypotheses for complex diseases. *Curr. Opin. Genet. Dev.* 19 212–219. 10.1016/j.gde.2009.04.010 19481926 PMC2914559

[B32] Shen X. Song S. Li C. Zhang J. (2022). Synonymous mutations in representative yeast genes are mostly strongly non-neutral. *Nature* 606 725–731. 10.1038/s41586-022-04823-w 35676473 PMC9650438

[B33] Sonney S. Leipzig J. Lott M. T. Zhang S. Procaccio V. Wallace D. C. (2017). Predicting the pathogenicity of novel variants in mitochondrial tRNA with MitoTIP. *PLoS Comput. Biol.* 13:e1005867. 10.1371/journal.pcbi.1005867 29227991 PMC5739504

[B34] Starikovskaya E. Shalaurova S. Dryomov S. Nazhmidenova A. Volodko N. Bychkov I. (2019). Mitochondrial DNA Variation of Leber’s Hereditary Optic Neuropathy in Western Siberia. *Cells* 8:1574. 10.3390/cells8121574 31817256 PMC6953113

[B35] van Oven M. (2015). PhyloTree Build 17: growing the human mitochondrial DNA tree. *Forensic Sci. Int. Genet. Suppl. Ser.* 5 e392–e394.

[B36] Van Oven M. Kayser M. (2009). Updated comprehensive phylogenetic tree of global human mitochondrial DNA variation. *Hum. Mutat.* 30 E386–E394. 10.1002/humu.20921 18853457

[B37] Venter M. Malan L. Van Dyk E. Elson J. L. Van der Westhuizen F. H. (2017). Using MutPred derived mtDNA load scores to evaluate mtDNA variation in hypertension and diabetes in a two-population cohort: the SABPA study. *J. Genet. Genomics* 44 139–149. 10.1016/j.jgg.2016.12.003 28298255

[B38] Venter M. Tomas C. Pienaar I. S. Strassheim V. Erasmus E. Ng W.-F. (2019). MtDNA population variation in Myalgic encephalomyelitis/Chronic fatigue syndrome in two populations: a study of mildly deleterious variants. *Sci. Rep.* 9:2914. 10.1038/s41598-019-39060-1 30814539 PMC6393470

[B39] Wallace D. C. (2013). Bioenergetics in human evolution and disease: implications for the origins of biological complexity and the missing genetic variation of common diseases. *Philos. Trans. R. Soc. B Biol. Sci.* 368:20120267. 10.1098/rstb.2012.0267 23754818 PMC3685467

[B40] Wei W. Gomez-Duran A. Hudson G. Chinnery P. F. (2017). Background sequence characteristics influence the occurrence and severity of disease-causing mtDNA mutations. *PLoS Genet.* 13:e1007126. 10.1371/journal.pgen.1007126 29253894 PMC5757940

[B41] Weissensteiner H. Forer L. Fuchsberger C. Schöpf B. Kloss-Brandstätter A. Specht G. (2016). mtDNA-Server: next-generation sequencing data analysis of human mitochondrial DNA in the cloud. *Nucleic Acids Res.* 44 W64–W69. 10.1093/nar/gkw247 27084948 PMC4987870

