# Supplementary material for: Mitochondrial DNA variation in Parkinson’s disease: Analysis of “out-of-place” population variants as a risk factor
Source: Front Aging Neurosci. 2022 Jul 14;14:921412. doi: 10.3389/fnagi.2022.921412 (PMC9330142; doi:10.3389/fnagi.2022.921412)
Supplement: Supplementary file 3 [file Table_3.DOCX]

Supplementary Material

# Supplementary Methods

# Next-generation sequencing (NGS) of African ancestry discovery cases and controls

## DNA isolation and quality control

# Genomic DNA (gDNA) was isolated from whole blood collected from participants of the previously-recruited African ancestry PD study cohort, using the NucleoSpin Blood XL kit (Macherey-Nagel Düren, Germany). Extracted gDNA was quantified on the Qubit 4.0 Fluorometer (Thermo Fisher Scientific, Waltham, USA). Spectrophotometry was performed on the NanoDrop® ND-1000 (Thermo Fisher Scientific) to determine the presence of any contaminants, i.e., protein contaminants in the gDNA samples. Genomic quality scores (GQS) were determined on the LabChip GXII Touch (Perkin Elmer, Waltham, USA). The GQS is a value between 0 and 5, with 0 indicating degraded gDNA and 5 intact gDNA. DNA that did not pass quality control was either replaced by another aliquot of DNA or was purified using the PureLink™ Genomic DNA Mini Kit (Thermo Fisher Scientific) according to the manufacturer’s protocol.

## Library and template preparation

# The Ion AmpliSeq™ Library Kit Plus (Thermo Fisher Scientific) was used to amplify target regions from 0.1 ng input gDNA. Targets were amplified across 21 cycles on the SimpliAmp thermal cycler (Thermo Fisher Scientific) using the Precision ID mtDNA Whole Genome Panel (Thermo Fisher Scientific) according to the manufacturer’s protocol. It is a 2-pool multiplex assay that targets the entire human mitochondrial genome. Each pool contains 81 primer pairs, with minimal primer overlap between pools. Following target amplification, IonCode™ Barcode Adapters (Thermo Fisher Scientific) were used to generate adapter-ligated libraries that were quantified using the Ion Library TaqMan Quantitation Kit (Thermo Fisher Scientific). qPCR amplification was performed using the StepOnePlus™ Real-time PCR system (Thermo Fisher Scientific).

# Libraries were diluted to a target concentration of 15 pM and loaded onto the Ion Chef liquid handler (Thermo Fisher Scientific) for template preparation. Subsequently, the templated library was loaded onto an Ion 530™ Chip (Thermo Fisher Scientific). NGS was performed on the Ion S5™ System (Thermo Fisher Scientific) according to the manufacturer’s protocol, using the Ion S5™ Sequencing Solutions and Reagents Kit (Thermo Fisher Scientific).

# Supplementary Results

The Sympathetic activity and Ambulatory Blood Pressure in Africans (SABPA) second control group, in which most individuals belonged to L0 maternal lineages, had the highest number of variants per person, whilst individuals from the replication study group belonging to European maternal lineages had the least (**Supplementary Table 3**). These findings were to be expected, as the phylogenetic distance between the rCRS (a European ancestry sequence belonging to haplogroup H2a2a1) and the African ancestry sequences is much greater than the distance between the European ancestry sequences and the rCRS. Overall, the mean number of variants per person (**Supplementary Table 3**) was similar between the cases and the controls from the discovery group, and between the cases and controls from the Parkinson’s disease Progression Markers Initiative (PPMI) replication group. This was to be expected, given that the cases and controls from these groups did not differ significantly in terms of maternal ancestry. Only the SABPA controls had higher variant counts per person compared to the discovery group cases. Again, this was expected due to the majority of the SABPA controls belonging to the deeply divergent L0 haplogroups. Interestingly, while the SABPA individuals had the highest number of haplogroup-defining variants (as expected), they also had the least number of non-haplogroup-defining variants.

|  | **Mean no. of variants per person (range)** | | | | |
| --- | --- | --- | --- | --- | --- |
| **Variant type** | **Discovery cases** | **Discovery controls** | **SABPA controls** | **PPMI cases** | **PPMI controls** |
| All variants | 61.7 ± 21.5 (31 – 90) | 61.4 ± 22.3 (28 - 89) | 71.1 ± 19.0 (31 – 91) | 24.9 ± 10.4 (8 – 42) | 25.0 ± 10.9 (7 – 40) |
|  |  |  |  |  |  |
| Haplogroup-defining | 59.0 ± 20.7 (29 – 88) | 59.2 ± 22.0 (26 – 88) | 69.1 ± 18.8 (29 – 90) | 22.7 ± 9.9 (6 – 39) | 22.7 ± 10.5 (6 – 39) |
|  |  |  |  |  |  |
| Non-haplogroup-defining | 2.7 ± 2.2 (0 – 10) | 2.3 ± 1.8 (0 – 11) | 1.98 ± 2.0 (0 – 9) | 2.2 ± 1.6 (0 – 8) | 2.4 ± 1.6 (0 – 8) |
|  |  |  |  |  |  |
| OXPHOS genes | 38.7 ± 14.5 (18 – 59) | 38.3 ± 15.2 (18 – 61) | 44.2 ± 12.4 (17 – 64) | 13.1 ± 6.2 (2 – 28) | 13.1 ± 6.4 (2 – 28) |
|  |  |  |  |  |  |
| Synonymous | 51.8 ± 18.1 (25 – 74) | 51.8 ± 19.0 (22 – 75) | 60.3 ± 15.8 (25 – 77) | 19.7 ± 8.5 (5 – 37) | 19.9 ± 8.9 (5 – 33) |
|  |  |  |  |  |  |
| Non-synonymous | 9.8 ± 3.8 (3 – 19) | 9.6 ± 3.7 (4 – 17) | 10.8 ± 3.5 (4 – 18) | 5.2 ± 2.4 (2 – 12) | 5.2 ± 2.5 (2 – 10) |
|  |  |  |  |  |  |
| Non-coding DNA | 12.8 ± 4.0 (6 – 25) | 13.1 ± 4.2 (6 – 25) | 15.0 ± 4.3 (7 – 26) | 6.9 ± 3.2 (1 – 16) | 7.1 ± 3.3 (12 – 15) |
|  |  |  |  |  |  |
| rRNA genes | 7.2 ± 1.9 (3 – 11) | 7.2 ± 2.1 (3 – 11) | 8.3 ± 1.8 (4 – 11) | 4.0 ± 1.3 (1 – 7) | 3.9 ± 1.2 (2 – 6) |
|  |  |  |  |  |  |
| tRNA genes | 3.0 ± 2.5 (0 – 7) | 3.5 ± 2.7 (0 – 7) | 3.5 ± 2.2 (0 – 7) | 1.0 ± 0.9 (0 – 3) | 0.9 ± 0.9 (0 – 3) |
|  |  |  |  |  |  |
| Transitions | 59.7 ± 19.9 (31 – 85) | 59.4 ± 20.7 (28 – 85) | 68.7 ± 17.4 (31 – 87) | 24.3 ± 10.0 (8 – 41) | 24.4 ± 10.5 (7 – 39) |
|  |  |  |  |  |  |
| Transversions | 2.0 ± 1.9 (0 – 6) | 1.9 ± 1.8 (0 – 6) | 2.4 ± 1.7 (0 – 6) | 0.6 ± 0.8 (0 – 4) | 0.6 ± 0.8 (0 – 5) |

**Supplementary Table 3.** Mean number of variants per person for each of the different study group cases and controls

PPMI, Parkinson’s Progression Markers Initiative; OXPHOS, oxidative phosphorylation; SABPA, Sympathetic activity and Ambulatory Blood Pressure in Africans study

**Supplementary Table 4.** Haplogroup distribution for each study group

|  |  | **Discovery group** | | **Second control group** | **Replication group** | |
| --- | --- | --- | --- | --- | --- | --- |
| **Ancestry** | **Haplogroups** | **No. cases (%)** | **No. controls (%)** | **No. SABPA controls (%)** | **No. PPMI cases (%)** | **No. PPMI controls (%)** |
| African |  |  |  |  |  |  |
|  | L0 | 28 (40) | 36 (46) | 35 (66) | - | - |
|  | L1 | 4 (6) | 3(4) | 1 (2) | - | - |
|  | L2 | 18 (26) | 15 (19) | 11 (21) | - | - |
|  | L3 | 20 (29) | 24 (31) | 6 (11) | - | - |
|  | L4 | 0 | 0 | 0 | - | - |
|  | L5 | 0 | 0 | 0 | - | - |
|  | L6 | 0 | 0 | 0 | - | - |
| European |  |  |  |  |  |  |
|  | H | - | - | - | 92 (33) | 49 (35) |
|  | I | - | - | - | 14 (5) | 4 (3) |
|  | J | - | - | - | 27 (10) | 13 (9) |
|  | K | - | - | - | 37 (13) | 20 (14) |
|  | T | - | - | - | 37 (13) | 17 (12) |
|  | U | - | - | - | 51 (18) | 24 (17) |
|  | V | - | - | - | 10 (4) | 5 (4) |
|  | W | - | - | - | 6 (2) | 5 (4) |
|  | X | - | - | - | 7 (2) | 3 (2) |

PPMI, Parkinson’s Progression Markers Initiative; SABPA, Sympathetic activity and Ambulatory Blood Pressure in Africans study

***** End of supplementary material *****
